# Endogenous SH2B1 protein localizes to lamellipodia and filopodia: platinum replica electron-microscopy study

**DOI:** 10.17912/micropub.biology.001451

**Published:** 2025-01-17

**Authors:** Maria Diakonova, Christin Carter-Su, Tatyana Svitkina

**Affiliations:** 1 Biological Sciences, University of Toledo, Toledo, Ohio, USA; 2 University of Michigan Medical School, Ann Arbor, Michigan, USA; 3 University of Pennsylvania, Philadelphia, Pennsylvania, USA

## Abstract

The widely expressed adapter protein SH2B1 was initially identified as a binding partner and substrate of tyrosine kinase JAK2. SH2B1β potentiates JAK2 activation in response to different ligands, including growth hormone, leptin and prolactin. SH2B1β has been implicated in cell motility and regulation of actin rearrangement in response to growth hormone, prolactin and platelet-derived growth factor. Here we use immunofluorescence and platinum replica electron-microscopy (PREM) technique to study localization of endogenous SH2B1. We show that endogenous SH2B localizes to two actin-rich protrusive organelles in cells: lamellipodia and filopodia. Based on this and previously published data, we suggest that at least some SH2B1 isoforms directly bind to actin filaments in both structures. Additionally, SH2B1 isoforms may work as a partner of filamin A in lamellipodia and VASP in filopodia participating in modulation of the actin cytoskeleton in response to extracellular signals

**Figure 1. Endogenous SH2B1 localizes to lamellipodia and filopodia:**
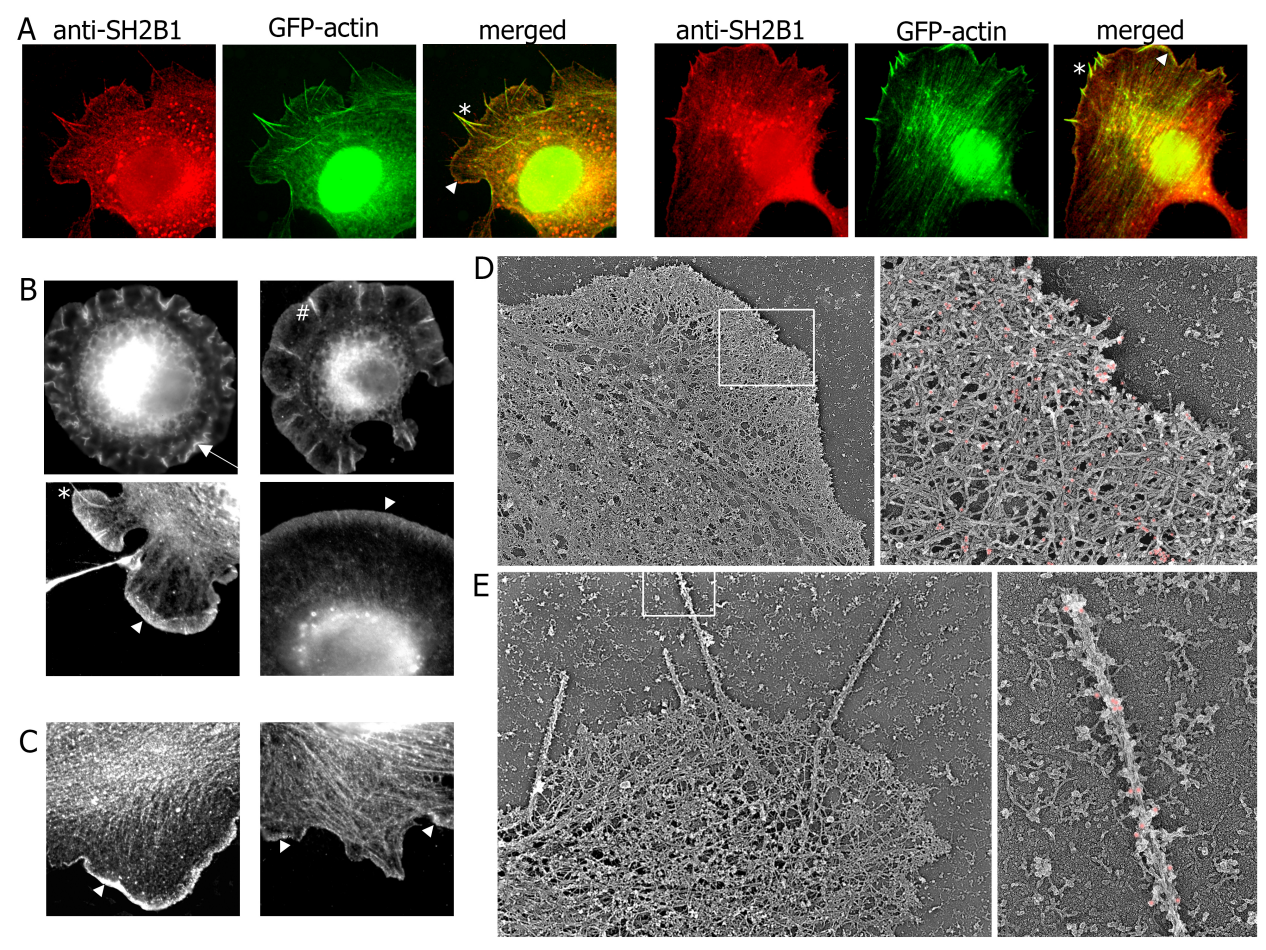
**A)** Melanoma B16-F1 cells stably overexpressing EGFP- actin were spread on laminin-coated coverslips, treated with Triton-X-100, fixed, and immunolabelled with anti-SH2B1 antibody followed by rabbit anti-goat -Texas Red antibody. Endogenous SH2B1 (red) co-localized with EGFP-actin (green) in both lamellipodia (arrowhead) and filopodia (asterisk) (merged images). **B - C)** Mouse 3T3-F442A fibroblasts were either spread on laminin-coated coverslips (B) or on plain coverslips (C) and treated as in A. Endogenous SH2B1 localized to cell ruffles (arrows), lamellipodia (arrowheads), microspike (pound sign) and filopodia (asterisk). **D - E)** Mouse 3T3-F442A fibroblasts were spread on laminin-coated coverslips, treated with Triton-X-100, fixed, immunolabeled with anti-SH2B1 antibody followed by rabbit anti-goat -colloidal gold-conjugated antibody and proceeded for PREM. Low magnification images demonstrate lamellipodia with a dense network of filaments (Panel D, left image) and long finer-like protrusions, filopodia (Panel E, left image). High resolution images demonstrate that SH2B1-immunogold particles (pseudo-colored in pink) are more abundant at the edge of lamellipodia (Panel D, right image) and distributed along the length of the filopodium (Panel E, right image). Panel E (right image) also shows that filopodia are composed of a bundle of long, unbranched filaments along the length of filopodia.

## Description

SH2B1 is an SH2 domain‐containing adapter protein participating in cell signaling in response to various growth factors, hormones, and cytokines, including growth hormone, prolactin, insulin, platelet-derived growth factor (PDGF), IGF-I, leptin, nerve growth factor, brain‐derived neurotrophic factor and fibroblast growth factor (reviewed in (Cheng et al., 2020; Desbuquois et al., 2013; Maures et al., 2007; Rui, 2014) ). Rare variants of SH2B1 in humans are associated with childhood obesity, insulin resistance, hyperphagia, short stature as adults and maladaptive behavior (Doche et al., 2012; Flores et al., 2019; Li et al., 2024; Pearce et al., 2014) . More recently, SH2B1 has been linked to the progression of cancer, including lung, esophageal, gastric, and oropharyngeal cancers (reviewed in (Cheng et al., 2020) ). Although SH2B1 is highly expressed in these cancers and serves as an indicator of poor prognosis, the exact function of SH2B1 in tumors is not yet clear. The SH2B1 gene encodes four isoforms (α, β, γ and δ) due to alternative mRNA splicing. These isoforms share identical dimerization, pleckstrin homology and SH2 domains but differ at their C termini (Nelms et al., 1999; Yousaf et al., 2001) . Notably, all SH2B1 isoforms also contain a nuclear localization sequence and a nuclear export sequence (Carter-Su et al., 2023) . In humans, the β and γ isoforms are expressed ubiquitously, whereas the α and δ isoforms are found predominantly in the brain (Doche et al., 2012) .

SH2B1β, one of the widely expressed isoforms, was initially identified as a binding partner and substrate of the tyrosine kinase JAK2 (Rui et al., 1997) . SH2B1β is recruited to phosphorylated tyrosine (Tyr) 813 in activated JAK2 and is in turn phosphorylated by JAK2 at Tyr(s) 439 and 494 (Kurzer et al., 2004; O'Brien et al., 2003; Rui et al., 1997). JAK2 phosphorylation of Tyr in SH2B1β suggests that other SH2 domain-containing signaling proteins may be recruited to GH receptor/JAK2/SH2B1 complexes. SH2B1 has been shown to enhance JAK2 activity (Rui & Carter-Su, 1999) , SH2B1β has been implicated in the regulation of the actin cytoskeleton. It modulates membrane ruffling, pinocytosis, lamellipodial activity and cell migration (Diakonova et al., 2002; Herrington et al., 2000) . Three proteins implicated in the regulation of the actin cytoskeleton have been identified as SH2B1 direct or indirect binding proteins – the small GTPas Rac1 (Diakonova et al., 2002) , the guanine nucleotide exchange factor Vav3 (Carter-Su et al., 2023) and bacterial ActA protein from *Listeria monocytogenes* which works as a functional homolog of eukaryotic WASP family proteins (Diakonova et al., 2007) . Interestingly, SH2B1β is essential for the maximal actin-based motility of the bacterium *Listeria monocytogenes* both *in vivo* and *in vitro,* through a vasodilator-stimulated phosphoprotein (VASP)-dependent mechanism (Diakonova et al., 2007) .

There are two actin-rich protrusive organelles in cells: lamellipodia and filopodia. Lamellipodia are flat and broad protrusions that are often seen in fast moving cells. Actin filaments in lamellipodia are organized in a network of branched filaments that undergo constant *de novo* nucleation (Borisy & Svitkina, 2000; Svitkina & Borisy, 1999; Svitkina et al., 1997) . Conversely, filopodia are actin-rich finger-like plasma membrane extensions that have been implicated in several cellular processes including cell-cell signaling, guidance toward chemoattractants, embryonic development, wound healing, metastasis, and adhesion to the extracellular matrix (for review (Gupton & Gertler, 2007; Mattila & Lappalainen, 2008) ). Filopodia contain a bundle of long actin filaments undergoing continuous barbed-end elongation at their tips (Mallavarapu & Mitchison, 1999; Small et al., 1978) . While lamellipodia and filopodia share some common actin-binding proteins, such as the Ena/VASP proteins, formins, cofilin, filamin A and profilin, they also have distinct proteins that provide their unique structural and functional needs. Thus, lamellipodia rely more on proteins involved in branched actin network assembly and stabilization, such as the Arp2/3 complex, WASP family nucleation-promoting factors, capping protein, and cortactin. Filopodia contain parallel, bundled actin filaments, so they require proteins that promote bundling and elongation, such as fascin and myosin X (Campellone & Welch, 2010; Mattila & Lappalainen, 2008; Pollard & Cooper, 2009; Svitkina & Borisy, 1999) .

We have previously demonstrated that SH2B1β localizes to the plasma membrane, cytoplasm, and membrane ruffles (Diakonova et al., 2002; Herrington et al., 2000) , along filopodia (Rider et al., 2009) and to focal adhesions (Lanning et al., 2011) ; it also cycles between the nucleus, the cytoplasm and the plasma membrane (reviewed in (Carter-Su et al., 2016) ). However, these localization studies relied on the overexpression of GFP- or myc-tagged forms of SH2B1β (Diakonova et al., 2002; Herrington et al., 2000; Lanning et al., 2011; Rider & Diakonova, 2011; Rider et al., 2009; Su et al., 2013) , and data on the localization of endogenous SH2B1 isoforms, especially in connection with the actin cytoskeleton, are lacking.

To study the localization of endogenous SH2B1, we used mouse melanoma B16-F1 cells stably expressing EGFP- actin because these cells form well-developed lamellipodia and filopodia on laminin-coated coverslips. We made cytoskeleton preparations from spread cells using Triton X-100 extraction in a cytoskeleton-preserving buffer. This approach preserves the cytoskeleton-associated fraction of SH2B1 while removing its cytosolic and membrane-associated subpopulations. After extraction, we fixed the samples and labeled them with primary anti-SH2B1 antibody followed by secondary Texas Red-conjugated antibody. The anti-SH2B1 antibody was made against a portion of SH2B1 that is shared by all isoforms. Fluorescence microscopy of these samples revealed the formation of large, thin lamella with broad actin-rich lamellipodia at the leading edge of the cell, as well as the formation of actin-rich filopodia ( Fig. 1 A, green). Endogenous SH2B1 ( Fig. 1A, red) co-localized with EGFP-actin in both lamellipodia ( Fig. 1A, arrowhead) and filopodia ( Fig. 1A, asterisk). To confirm these data in a different cell line, we used mouse 3T3-F442A fibroblasts, also spread on laminin-coated coverslips ( Fig. 1B ). In contrast to melanoma B16-F1 cells, 3T3-F442A fibroblasts rarely form conventional filopodia extending beyond the leading edge, but form related structures –microspikes, which are actin filament bundles embedded in lamellipodia. Lamellipodia in these cells under our culture conditions were distributed around the entire cell perimeter, resulting in the so-called “fried egg” phenotype (Applewhite et al., 2007) . Endogenous SH2B1 localized at the outer edge of the cells, with pronounced labeling of cell ruffles ( Fig. 1B, arrows), lamellipodia ( Fig. 1B, arrowhead), microspikes ( Fig. 1B, pound sign) and filopodia ( Fig. 1B, asterisk). Endogenous SH2B1 also localized to lamellipodia in 3T3-F442A cells spread on plain coverslips ( Fig. 1C, arrowhead).

Electron microscopy (EM) was carried out to evaluate the localization of endogenous SH2B1 in more detail. We used platinum replica electron-microscopy (PREM), which combines the high resolution of EM with an ability to preserve three-dimensionality of the cell (reviewed in (Svitkina, 2022) ). We made cytoskeleton preparations using Triton X-100 extraction, fixed the samples, labeled them with primary anti-SH2B1 antibody followed by secondary colloidal gold-conjugated antibody, and processed them for PREM. Low magnification views showed that the cytoskeleton of lamellipodia in 3T3-F442A cells consists of a dense network of actin filaments ( Fig. 1D ). High resolution analysis (upper right panel in Fig. 1D ) demonstrated the presence of Y-shaped actin filament branches within lamellipodia characteristic for the Arp2/3 complex–dependent actin array. Individual filaments could be observed primarily near the leading edge. At some cell edges, tight bundles of actin filaments crossed the lamellipodial network and extended beyond the leading edge forming long finger-like protrusions, which represent filopodia ( Fig. 1E ). As expected, the filopodium was composed of a bundle of long, unbranched actin filaments running parallel to each other along the length of the filopodium ( Fig. 1E, lower right panel). In lamellipodia, SH2B1-immunogold particles (highlighted in pink in Fig. 1D, right panel) were more abundant at the edge compared with more internal lamellipodial regions. Consistent with the immunofluorescence data, SH2B1-immunogold particles were present along the length of filopodium ( Fig. 1E, right image).

Thus, we have demonstrated by immunofluorescence and PREM that endogenous SH2B1 localizes to two highly dynamic protrusive organelles in cells: lamellipodia and filopodia. Both of these structures are enriched with actin filaments. Since we have previously shown that both N-terminal amino acids (150-200) shared by all SH2B1 isoforms and C-terminal amino acids (615-670) of SH2B1β bind to F-actin *in vitro* (Rider et al., 2009) , we speculate that SH2B1β and most likely other SH2B1 isoforms participate in the organization of the actin cytoskeleton in both lamellipodia and filopodia. We have also previously shown that amino acids 200-260 of SH2B1 isoforms interact directly with the repeats 17-23 (amino acids 1863-2522) of actin-binding protein filamin A (FLNa) (Rider & Diakonova, 2011) . Another protein of the SH2B family, SH2B3 (or Lnk), was identified in a yeast two-hybrid screen as a partner for FLNa (He et al., 2000) . Filamin A cross-links actin filaments, contributing to the dense, mesh-like structure characteristic of lamellipodia. The role of filamin A in membrane ruffles and lamellipodia formation is defined by its actin binding properties, its ability to rearrange three-dimensional actin networks, as well as its capacity to initiate protein–protein interactions (reviewed in (Lamsoul et al., 2020; Shead et al., 2024) ). Thus, we speculate that SH2B1β (and perhaps other SH2B1 isoforms) may regulate the actin cytoskeleton in lamellipodia via its direct binding to actin and FLNa.

Another SH2B1 partner that likely plays a critical role in SH2B1 regulation of the actin cytoskeleton is VASP. In contrast to FLNa, actin-binding protein VASP promotes actin filament elongation and prevents capping, aiding in the formation of long, parallel filaments more typical for filopodia than for lamellipodia, although it also promotes lamellipodia protrusion. We have previously found that SH2B1β localization in filopodia depends on the first actin-binding site of SH2B1β and the presence of VASP (Rider et al., 2009) . Thus, wild-type SH2B1β overexpressed in 3T3-F442A cells localized along filopodia, but deletion of amino acids 150–200 (the first actin-binding site) led to mislocalization of the protein to filopodia “tip complexes” where it colocalized with VASP. Localization of SH2B1β in filopodia was also dramatically changed in MVD7 -/- cells generated from VASP/Mena double-knockout mice. This effect was reversible; after the re-expression of VASP in MVD7 -/- cells, SH2B1β again localized along the length of filopodia (Rider et al., 2009) . Additionally, overexpression of SH2B1β increases the number of IRSp53-induced filopodia (Hong et al., 2014) .

Interestingly, SH2B1β localizes to *Listeria* -induced actin tails. Bacterial motility is increased in infected cells overexpressing SH2B1β, increased in *Xenopus* egg extracts supplemented with GST- SH2B1β and decreased in mouse embryo fibroblasts from SH2B -/- mice. Both the recruitment of SH2B1β to *Listeria* and SH21Bβ stimulation of actin-based propulsion require VASP, which binds to bacterial protein ActA at the surfaces of *Listeria* cells and enhances bacterial actin-based motility (Diakonova et al., 2007) .

In conclusion, we have demonstrated here that endogenous adaptor protein SH2B1 localizes in two actin-rich protrusive organelles in cells: lamellipodia and filopodia. Based on the results presented here and previously published data, we speculate that SH2B1 may act to assemble a multiprotein signaling complex to couple upstream activators (cytokines and cytokine receptors) to downstream effectors (actin cytoskeleton) to regulate the actin cytoskeleton and the cytokine-dependent motility in normal and tumor cells.

## Methods

**Cell culture**

The mouse melanoma B16F1 cell line stably expressing EGFP-β-actin was provided by Dr. C. Ballestrem (Weizmann Institute of Science, Rehovot, Israel) and cultured as described previously (Ballestrem et al., 1998) . The original stock of mouse 3T3-F442A fibroblasts was provided by H. Green (Harvard University, Cambridge, MA) and cultured as described previously (Herrington et al., 2000) .

**Immunofluorescence**

The cells were plated onto coverslips coated with 10–25 µg/ml laminin and blocked with 0.1 µg/ml heat-inactivated BSA. Immunostaining was performed after cell extraction for 3–10 min with 1% Triton X-100 in PEM buffer (100 mM Pipes, pH 6.9, 1 mM MgCl2, and 1 mM EGTA), containing 4% polyethylene glycol, (mol wt 40,000), and 2 µM phalloidin, followed by fixation with 0.2% glutaraldehyde and quenching with NaBH4 (2 mg/ml). After blocking of non-specific staining in 2% goat serum, the coverslips were incubated with anti-SH2B1 antibody followed by rabbit anti-goat -Texas Red antibody. Staining by secondary antibody alone was negligible. Light microscopy was performed using an inverted microscope (Eclipse; Nikon) equipped with a Plan 100X, 1.3 NA objective and a back-illuminated cooled CCD camera (model CH250; Roper Scientific) driven by MetaMorph® imaging software (Universal Imaging Corp.).

**Platinum replica electron-microscopy (PREM)**

3T3-F442A cells were plated onto coverslips coated with 10–25 µg/ml laminin and processed as described above. The coverslips were incubated with anti-SH2B1 antibody followed by rabbit anti-goat -colloidal gold- conjugated antibody. Samples were then fixed with tannic acid and uranyl acetate, critical point dried, coated with platinum and carbon, transferred onto electron microscopic grids, and analyzed using JEM 1011 transmission EM (JEOL USA) operated at 100 kV (Svitkina & Borisy, 1998) . Images were captured with an ORIUS 832.10W CCD camera (Gatan) and are presented in inverted contrast. Gold particles in PREM samples were identified at 200,000X magnification after contrast enhancement to distinguish them from other bright objects. Gold particles were pseudocolored using the Adobe Photoshop brush tool with 50% opacity.

## Reagents

Goat polyclonal anti-SH2B1 antibody raised against a peptide mapping within an internal region of SH2B1 of rat origin (E-20, sc-10827) was from Santa-Cruz Biotechnology. Rabbit anti-goat IgG - Texas Red was from Molecular Probes, Inc. Rabbit anti-goat IgG - 12 nm colloidal gold-conjugated antibody was from Jackson ImmunoResearch. Phalloidin was from Sigma-Aldrich, laminin from Invitrogen, and polyethylene glycol (mol wt 40,000) from Serva.
